# Perceived Contraceptive Counseling Quality Among Veterans Using VA Primary Care: Data from the ECUUN Study

**DOI:** 10.1007/s11606-022-07586-2

**Published:** 2022-08-30

**Authors:** Lisa S. Callegari, Siobhan S. Mahorter, Sam K. Benson, Xinhua Zhao, Eleanor Bimla Schwarz, Sonya Borrero

**Affiliations:** 1 Health Services Research and Development, Department of Veterans Affairs (VA) Puget Sound Health Care System, 1660 S. Columbian Way S-152, Seattle, WA 98108 USA; 2 Department of Obstetrics & Gynecology, University of Washington School of Medicine, Seattle, USA; 3 Department of Health Services, University of Washington School of Public Health, Seattle, USA; 4 Center for Health Equity, Research, and Promotion, VA Pittsburgh Health Care System, Pittsburgh, USA; 5 Department of Medicine, University of California, Davis, Sacramento, CA USA; 6 Department of Medicine, University of Pittsburgh School of Medicine, Pittsburgh, USA

**Keywords:** patient-centered care, provider-patient communication, contraceptive counseling, women Veterans, primary care

## Abstract

**Background:**

High-quality contraceptive counseling is critical to support Veterans’ reproductive autonomy and promote healthy outcomes.

**Objective:**

To describe perceived quality of contraceptive counseling in Veterans Health Administration (VA) primary care and assess factors associated with perceived high- and low-quality contraceptive counseling.

**Design:**

Cross-sectional study using data from the Examining Contraceptive Use and Unmet Need in women Veterans (ECUUN) national telephone survey.

**Participants:**

Veterans aged 18–44 who received contraceptive services from a VA primary care clinic in the past year (*N*=506).

**Main Measures:**

Perceived quality of contraceptive counseling was captured by assessing Veterans’ agreement with 6 statements regarding provider counseling adapted from the Consumer Assessment of Healthcare Providers and Systems (CAHPS) survey. High-quality counseling was defined as a top score of strongly agreeing on all 6 items; low-quality counseling was defined as not agreeing (neutral, disagreeing, or strongly disagreeing) with >3 items. We constructed two multivariable models to assess associations between patient-, provider-, and system-level factors and perceived high-quality (Model 1) and perceived low-quality counseling (Model 2).

**Key Results:**

Most participants strongly agreed that their providers listened carefully (74%), explained things clearly (77%), and spent enough time discussing things (71%). Lower proportions strongly agreed that their provider discussed more than one option (54%), discussed pros/cons of various methods (44%), or asked which choice they thought was best for them (62%). In Model 1, Veterans who received care in a Women’s Health Clinic (WHC) had twice the odds of perceiving high-quality counseling (aOR=1.99; 95%CI=1.24–3.22). In Model 2, Veterans who received care in a WHC (aOR=0.49; 95%CI=0.25–0.97) or from clinicians who provide cervical cancer screening (aOR=0.49; 95%CI=0.26–0.95) had half the odds of perceiving low-quality counseling.

**Conclusions:**

Opportunities exist to improve the quality of contraceptive counseling within VA primary care settings, including more consistent efforts to seek patients’ perspectives with respect to contraceptive decisions.

## INTRODUCTION

Veterans who are capable of pregnancy are one of the fastest-growing and most diverse groups of patients served by the Veterans Health Administration (VA).^1^ Among the population of over 200,000 Veterans aged 18–44 identified as women in VA administrative data, nearly half (48%) belong to a minoritized racial or ethnic group.^1^ Furthermore, this population has complex medical and mental health needs, as well as a high prevalence of psychosocial stressors such as homelessness, intimate partner violence, and sexual trauma, including military sexual trauma (MST).^1–4^

Providing reproductive health services that meet the needs of this population is a high priority for VA.^5^ Contraceptive counseling is one of the most commonly needed health services for people capable of pregnancy, including Veterans.^1^ With fewer than 10% of Veterans who are able to become pregnant actively seeking pregnancy,^6^ high-quality contraceptive counseling and care are critical to ensure Veterans can avoid unwanted pregnancy and achieve their family formation desires. National guidelines from the Centers for Disease Control and Prevention and Office of Population Affairs outline key components of high-quality contraceptive counseling and care, which include recommendations that contraceptive care is provided in a patient-centered manner and that people have access to information about the full range of contraceptive options available.^7^

Patient-centered care, as defined by the National Academies of Medicine, is care that is respectful of and responsive to individual patient preferences, needs, and values and ensures that patient values guide all clinical decisions.^8^ Patient-centered counseling is particularly important for contraception, which is a preference-sensitive decision in which people have multiple options and the best option depends on individuals’ assessment of the relative importance of different characteristics or potential outcomes.^9^ Studies demonstrate that people’s preferences about contraception vary widely, such as how important it is that a method is effective at preventing pregnancy or whether or not a method contains hormones.^10, 11^ Furthermore, explicitly prioritizing preferences, needs, and values in communication about contraception is particularly critical for low-income people and Black, Indigenous, and other people of color, given the USA’s long history of abuses of reproductive autonomy through coercive practices and policies related to contraception and sterilization in those populations.^12^

High-quality contraceptive care begins with treating each person with respect, empathy, and understanding and building a trusting relationship.^13^ Essential to the process is eliciting individual patients’ values and preferences regarding attributes of contraceptive methods and then offering easy to understand, evidence-based information about methods that best align with their stated preferences.^13^ Best practices also include offering information about the range of options that are medically safe for a person^7, 14^; this recommendation is relevant in contraception because people may have varying knowledge about their options and providers often fail to offer information about multiple methods or to help patients understand the relevant benefits and side effects of a range of methods in counseling encounters.^7^ However, in some cases, people may prefer to discuss only one method or make their decision with limited input from providers.^15^ High-quality counseling includes offering information but allows patients to express their preferences about how much information to receive and the extent of provider involvement in the decision-making process.^15, 16^

With the growing population of Veterans capable of pregnancy relying on VA for care, ensuring high-quality contraceptive care will continue to be a priority.^5^ To date, few data are available to inform efforts to address gaps in contraceptive counseling quality in VA. While qualitative studies highlight some Veterans’ negative experiences with family planning counseling within VA,^17–19^ no population-level data describes Veterans’ experiences with contraceptive counseling within VA or factors associated with quality of care. We examined national data from the Examining Contraceptive Use and Unmet Need in women Veterans (ECUUN) study^20^ to describe quality of contraceptive counseling in VA primary care and to assess patient-, provider-, and system-level factors associated with Veterans’ experiences of high- and low-quality counseling in VA.

## METHODS

### Study Population and Sample

We analyzed data from the ECUUN study, a national, cross-sectional telephone survey of Veterans ages 18-44 who were identified as women in VA administrative data and had received primary care at VA in the past year.^20^ Of note, ECUUN excluded individuals not assigned female sex at birth but did not further assess gender identity in screening or in the survey; therefore, the sample may have also included individuals who did not identify as women and were miscoded in the administrative data. The survey was administered between April 2014 and January 2016 and assessed Veterans’ current contraceptive use, pregnancy history, experiences receiving primary care at VA, health conditions, and demographic characteristics. Detailed survey methodology is described elsewhere.^20^ The ECUUN study was approved by both the VA Pittsburgh and University of Pittsburgh institutional review boards.

This analysis included Veterans who reported that they received contraceptive counseling from their VA primary care provider (PCP) in the past year. Veterans who reported a prior history of hysterectomy or infertility or who did not complete all contraceptive quality questions were excluded.

### Measures

#### Patient-Reported Experiences of Contraceptive Counseling

The ECUUN survey measured Veterans’ perceptions of contraceptive counseling quality using six items with 5-point Likert-scale response options. Items were adapted from the Consumer Assessment of Healthcare Providers and Systems (CAHPS) Patient-Centered Medical Home Version 2.0 survey questions.^21^ These questions included asking about an agreement that a PCP “listened carefully to your questions and/or concerns about contraception,” “explained things in a way that was easy to understand,” “spent enough time discussing things with you,” and “asked you which contraceptive choice you thought was best for you.” Two additional questions assessed whether information about more than one contraceptive option was discussed, asking about an agreement that a PCP “talked about more than one type of contraception option” and “talked to you about pros and cons of various types of contraceptive methods.” Of note, these two questions are phrased in such a way that a negative response could still represent patient-centered care if a patient preferred to receive information only about one option. However, given that providing information about multiple options is a best practice in many cases, these items are included as an approximation of best practices.

Item response options were “strongly agree,” “agree,” “neutral,” “disagree,” and “strongly disagree.” Given that responses to healthcare experience surveys tend to be skewed with the majority of responses at the positive end of the scale,^22^ prior studies of provider-patient communication quality have used the proportion receiving top scores (i.e., most positive versus all other responses) to characterize high-quality healthcare experiences.^23–26^ Prior work also underscores the importance of examining negative responses, which may have a greater ability to discriminate differences in patient experience. Given recommendations that cut-points should be selected so that negative responses comprise 10–15% of the sample,^27, 28^ we created two binary variables: (1) high-quality counseling defined as a top score of “strongly agree” to all six items; (2) low-quality counseling defined as less than “agree” or “strongly agree” (i.e., responses of “neutral,” “disagree,” or “strongly disagree”) to more than half of the items.

#### Patient-, Provider-, and System-Level Factors

We selected factors that we hypothesized could be associated with perceived contraceptive counseling quality. All variables were self-reported in the ECUUN survey except for geographic region, which was ascertained from VA administrative data.

Patient-level factors included age, marital status, education, annual household income, history of one or more medical conditions that might affect contraceptive counseling (hypertension, history of thromboembolic disease, breast cancer, stroke, liver disease, HIV/AIDS, obesity, diabetes, migraines, systemic lupus erythematosus, seizure disorders, or tobacco use), history of one or more mental health conditions (major depression, bipolar, post-traumatic stress disorder, schizophrenia, or anxiety/panic disorder), history of MST, and parity. Mental health conditions and history of MST were assessed separately and collapsed into a single variable (both, one, neither) to capture the additive effect of mental health and MST.^29, 30^ Race/ethnicity and sexual orientation (gay/bisexual/asexual or heterosexual/straight) were included as social factors associated with poorer quality healthcare due to racism and/or discrimination.^31^

Provider-level factors included whether the Veteran sees their PCP for all or almost all of their medical care, whether they see their PCP for routine gynecologic care such as Pap smears (an indicator of clinician comfort providing sex-specific care), and PCP gender. Facility-level factors included geographic region (Northeast, Midwest, South, West) and a single variable capturing patient’s report of whether their VA facility includes a Women’s Health Clinic (WHC), and, if so, whether they received their care there (no WHC at site or don’t know, WHC at site but not seen there, WHC at site and seen there). A WHC is a specialized clinic designed to provide comprehensive care to women Veterans including both primary care and gender-specific care.^32^

### Statistical Analysis

We calculated frequencies and percentages to describe the study population and their experience of contraceptive counseling quality. Unadjusted associations between patient-, provider-, and system-level factors and high- and low-quality contraceptive counseling were calculated with chi-squared tests. Adjusted ORs and 95% CIs were estimated with separate multiple logistic regression models to evaluate associations between those factors and high-quality (Model 1) and low-quality (Model 2) contraceptive counseling. In addition to patient age and race/ethnicity which were forced into the models, factors associated with low-quality or high-quality contraceptive counseling at the *p*≤0.15 level were included in multivariate analyses. All analyses were conducted using SAS version 9.4 software (SAS Institute Inc., Cary, NC, USA).

## RESULTS

### Participants

Out of 2302 Veterans who completed the ECUUN survey, 543 had received contraceptive counseling or care from their VA PCP in the past year. Of those, 4 were excluded due to missing data on one or more questions about contraceptive counseling quality, and 33 were excluded due to a history of hysterectomy or infertility, yielding a final sample of 506 Veterans. The mean age of the sample was 32.8 (SD 5.3). Half were non-Hispanic White; 29% identified as non-Hispanic Black; 14% identified as Hispanic; and 7% identified as other race/ethnicity (Table 1). Over half of the participants had one or more medical conditions (56%). About 10% had a history of MST without a history of any mental health conditions, 23% had a history of one or more mental health conditions without a history of MST, and nearly half (44%) had a history of both.

**Table 1:** Characteristics of Veterans Who Received Contraceptive Counseling or Care at VA, 2014–2016*

Characteristic	*N* (%)
*Patient-level*	
Age (years)	
20–29	146 (28.9)
30–34	175 (34.6)
35–39	114 (22.5)
40–44	71 (14.0)
Race/ethnicity	
Non-Hispanic White	257 (50.8)
Non-Hispanic Black	146 (28.9)
Hispanic	70 (13.8)
Other	34 (6.5)
Sexual orientation	
Gay, bisexual or asexual	31 (6.2)
Heterosexual or straight	473 (93.8)
Marital status	
Single	143 (28.3)
Married	165 (32.6)
Living with partner	47 (9.3)
Divorced, separated, or widowed	151 (29.8)
Education	
High school or technical school	38 (7.5)
Some college	202 (39.9)
Bachelor’s degree or higher	266 (52.6)
Household income	
<$20,0000	100 (19.9)
$20,000–<$40,000	189 (37.6)
$40,000–<$60,000	99 (19.7)
≥$60,000	115 (22.9)
≥1 Medical condition	282 (55.7)
≥1 Mental health condition and/or MST	
Neither	120 (23.7)
Mental health condition only	115 (22.7)
MST only	48 (9.5)
Both mental health condition and MST	223 (44.1)
Parity ≥1	289 (57.1)
*Provider-level*	
Sees VA PCP for almost all care	441 (88.0)
Sees VA PCP for gynecologic care/Pap smears	396 (79.0)
VA PCP is female	429 (85.1)
*Facility-level*	
Geographic census region	
Northeast	51 (10.1)
Midwest	96 (19.0)
South	247 (48.8)
West	112 (22.1)
Primary care in VA Women’s Health Clinic (WHC)	
No WHC at site, or don’t know	129 (25.5)
WHC at site, not seen in WHC	68 (13.4)
WHC at site and seen in WHC	309 (61.1)

*MST* military sexual trauma; *PCP* primary care physician; *WHC* Women’s Health Clinic

*All participants received care from VA in the prior 12 months

### Quality of Contraceptive Counseling

**Figure 1:**
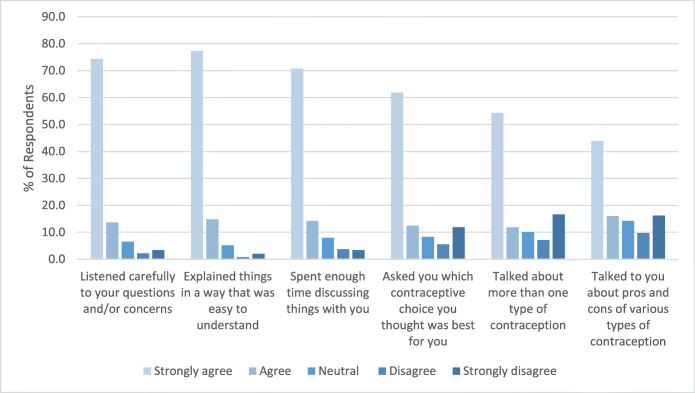
Veterans’ responses to six Likert-scale questions assessing the extent to which their VA primary care provider delivered components of high-quality contraceptive counseling (*N*=506).

Figure 1 presents the distribution of responses to each contraceptive counseling quality question. Most Veterans strongly agreed with statements that their provider listened to their questions and/or concerns about contraception carefully (74%), explained things in a way that was easy to understand (77%), and spent enough time discussing things with them (71%), with only a minority disagreeing or neutral (12%, 8%, 15%, respectively). Lower proportions of participants strongly agreed that their provider asked which contraceptive choice they thought was best for them (62%), discussed more than one type of contraceptive option (54%), or discussed the pros and cons of various methods (44%). Overall, 52% of Veterans (*n*=262) disagreed with or were neutral to one or more of the six statements. Using our definitions of high- and low-quality counseling, 32% reported high-quality counseling and 11% reported low-quality counseling.

#### Factors Associated with High-Quality Contraceptive Counseling

In bivariate analyses, a larger proportion of those receiving care at a WHC versus other locations reported high-quality counseling (Table 2). In the adjusted analysis including age and race/ethnicity (Model 1), Veterans seen in a WHC had twice the odds of reporting high-quality counseling compared to those who received primary care at a site with no WHC or who did not know (OR=1.99; 95%CI=1.24–3.22).

**Table 2:** Bivariate and Adjusted Associations Between Patient-, Provider-, and Facility-Level Characteristics and Contraceptive Counseling Quality Among Veterans Who Received Contraceptive Counseling, 2014–2016

	Model 1: Correlates of high-quality contraceptive counseling*			Model 2: Correlates of low-quality contraceptive counseling*		
	**% reporting high-quality contraceptive counseling**	***p*-value**^†^	**Adjusted OR (95% CI)**^‡^	**% reporting low-quality contraceptive counseling**	***p*-value**^†^	**Adjusted OR (95% CI)**^‡^
**Total**	**164 (32.4%)**			**55 (10.9%)**		
***Patient-level factors***						
**Age (years)**		0.71			0.12	
20–29	28.8		Ref	11.0		Ref
30–34	33.7		1.28 (0.79,2.09)	10.3		1.02 (0.47,2.22)
35–39	35.1		1.33 (0.78,2.28)	7.0		0.67 (0.26,1.71)
40–44	32.4		1.18 (0.63,2.19)	18.3		1.88 (0.78,4.50)
**Race/ethnicity**		0.39			0.32	
Non-Hispanic white	33.1		Ref	11.3		Ref
Non-Hispanic Black	32.2		0.84 (0.54,1.32)	7.5		0.72 (0.33,1.59)
Hispanic	25.7		0.69 (0.38,1.25)	15.7		1.61 (0.73,3.55)
Other	42.4		1.47 (0.69,3.10)	12.1		1.42 (0.43,4.64)
**Sexual orientation**		0.97			0.12	
Heterosexual	32.6			10.4		Ref
Gay/bisexual/asexual	32.3			19.4		1.80 (0.61,5.29)
**Marital status**		0.21			0.66	
Single	25.9			12.6		
Married	37.0			8.5		
Living with partner	31.9			10.6		
Divorced, separated or widowed	33.8			11.9		
**Education**		0.49			0.95	
High school or technical school	34.2			10.5		
Some college	35.1			10.4		
Bachelor’s degree or higher	30.1			11.3		
**Household income**		0.81			0.95	
<$20,0000	32.0			12.0		
$20,000–<$40,000	34.9			10.1		
$40,000–<$60,000	29.3			10.1		
≥$60,000	32.2			11.3		
**Medical conditions (including smoking)**		0.62			0.45	
No	31.3			12.1		
Yes	33.3			9.9		
**Mental health condition and/or MST**		0.74			0.02	
Neither	30.0			7.5		Ref
Either one	34.4			7.4		1.11 (0.42,2.92)
Both	32.3			15.2		2.14 (0.92,4.96)
**Parity**		0.52			0.20	
No	30.9			12.9		
Yes	33.6			9.3		
***Provider-level factors***						
**Patient sees PCP for all medical care**		0.18			0.04	
No	25.0			18.3		Ref
Yes	33.6			9.5		0.63 (0.29,1.36)
**Provider performs Pap smears**		0.79			0.002	
No	31.4			19.0		Ref
Yes	32.8			8.3		0.49 (0.26,0.95)
**Female provider**		0.95			0.43	
No	32.0			13.3		
Yes	32.4			10.3		
***Facility-level factors***						
**Geographic census region**		0.86			0.59	
Northeast	37.3			5.9		
Midwest	32.3			12.5		
South	32.4			10.5		
West	30.4			12.5		
**Primary care in VA Women’s Health Clinic (WHC)**		0.02			0.02	
No WHC at site, or don’t know	23.3		Ref	15.5		Ref
WHC at site, not seen in WHC	29.4		1.49 (0.76,2.92)	16.2		0.73 (0.30,1.80)
WHC at site and seen in WHC	36.9		1.99 (1.24,3.22)	7.8		0.49 (0.25,0.97)

*MST* military sexual trauma; *PCP* primary care physician; *WHC* Women’s Health Clinic

*High-quality contraceptive counseling defined as *strongly agree* with all 6 contraceptive counseling quality statements. Low-quality contraceptive counseling was defined as *neutral*, *disagree*, or *strongly disagree* to >3 statements

^†^Chi-squared test for bivariate association

^‡^In addition to patient age and race/ethnicity, factors associated with low-quality or high-quality contraceptive counseling at the *p*≤0.15 level were included in multivariate analyses

#### Factors Associated with Low-Quality Contraceptive Counseling

In bivariate analyses, smaller proportions of those who saw their PCP for all/most medical care, saw a PCP who provides Pap smears, and received care in a WHC reported low-quality counseling, while a larger proportion of those with a history of mental health condition(s) and/or MST reported low-quality counseling (Table 2). In the adjusted model (Model 2) which also included sexual orientation (*p*=0.12), age, and race, Veterans who received care in a WHC (OR=0.49; 95%CI=0.25–0.97) or saw a provider who performs Pap smears (OR=0.49; 95%CI=0.26–0.95) had about half the odds of reporting low-quality counseling compared to those who did not. Veterans with a history of mental health condition(s) and/or MST tended to be more likely to report low-quality contraceptive counseling compared to those with neither (OR=2.14; 95%CI=0.92–4.96).

## DISCUSSION

In this national sample of 506 Veterans who received contraceptive care from VA clinicians, we identified opportunities for improvement in contraceptive counseling quality, including more consistent efforts to seek patients’ perspectives with respect to contraceptive decisions. Provider- and system-level factors, such as care from PCPs who provide routine gynecologic care and being seen in a WHC, emerged as important predictors of counseling quality. While patient-level factors such as race/ethnicity and income were not correlated with counseling quality, Veterans with a history of mental health disorders and MST tended to perceive lower-quality counseling. These results can be used to inform policy and practice efforts to address existing gaps and improve the quality of contraceptive counseling within VA.

Although several qualitative studies highlight gaps in contraceptive counseling quality within VA,^17–19^ this is the first study to provide quantitative data from a national sample on Veterans’ perceptions of the contraceptive counseling they receive within VA primary care. Consistent with other studies of Veterans’ patient experience,^22, 23, 33^ most responses were positive with the majority of respondents agreeing or strongly agreeing with each statement. Nevertheless, our data highlight potential areas for improvement. For example, only three-quarters of Veterans strongly agreed that their PCPs listened carefully to their concerns and/or questions about contraception, a critical component of high-quality and patient-centered counseling. In addition, incorporating patients’ views about what method they think is best for them is a core requirement for patient-centered counseling; however, less than two-thirds of Veterans strongly agreed that their providers elicited this information. Our finding that only half of Veterans strongly agreed that their providers discussed more than one option or the pros and cons of multiple options may also indicate an opportunity for improvement; however, it is not possible to assess the extent to which this finding might reflect some Veterans’ preference to discuss only one option.^13, 34^

PCP proficiency with reproductive healthcare services and system-level factors such as receiving care at a WHC were associated with perceptions of high-quality contraceptive counseling. The VA has made substantial organization-wide efforts to improve the quality of care for women Veterans, including providing specialized training for PCPs, requiring that all women Veterans have access to a women’s health-trained PCP^32^ and provision of comprehensive primary care for women through creation of WHCs.^35^ These strategies have been demonstrated to be correlated with positive experiences of care and satisfaction more broadly,^33, 36^ and our data suggest that these strategies may also improve experiences of contraceptive counseling quality. In addition to continuing such investments, our study suggests that training on high-quality communication in the specific context of contraceptive counseling could also be helpful. Furthermore, novel interventions which empower Veterans to communicate their preferences and values in clinical encounters, such as patient-facing decision aids and communication tools, may also present opportunities to improve counseling interactions in VA.^37^

Although multiple studies outside VA highlight associations between poorer quality contraceptive care and social factors such as minoritized race/ethnicity and poverty,^38–40^ we did not identify these associations in our sample. Studies within VA have found worse patient experiences reported by Black and Latino Veterans,^27, 41^ including data from the ECUUN study demonstrating that more than 11% of Black and Latina Veterans reported race or ethnicity-based discrimination when seeking VA healthcare and that this discrimination was associated with their contraceptive choices.^42^ Additional studies designed specifically to investigate contraceptive care experiences of racial/ethnic subgroups of Veterans could help to explore this unexpected finding.

Veterans in our sample with mental health conditions and a history of MST had a non-significant trend towards perceiving lower-quality contraceptive counseling. This trend is consistent with studies in VA that have found individuals with mental health disorders and a history of MST are less likely to be satisfied with VA healthcare and more likely to report negative experiences.^43, 44^ Prior qualitative work by our group found that women Veterans with mental health disorders such as post-traumatic stress disorder perceived dismissal and devaluation of their concerns in contraceptive encounters^17^ and women with MST felt that their providers did not adequately address their concerns regarding contraceptive methods that require a pelvic exam and procedure.^17^ In light of our findings as well as ECUUN data demonstrating that Veterans with mental health conditions are more likely to experience unwanted or mistimed pregnancy compared to those without those conditions,^45^ additional efforts are indicated to better understand and address this population’s contraceptive counseling needs.

Strengths of this study included a large, national sample and inclusion of variables that are incompletely reported in VA administrative data, such as income, education, parity, and sexual activity. Limitations of this study include the possibility of recall bias, as the questions could have occurred up to a year after receipt of counseling, and reliance on self-report for provider- and system-level factors such as receipt of care in a WHC. In addition, the survey items used in ECUUN have not been validated specifically for contraceptive counseling. Since fielding of the ECUUN study, a measure of quality in interpersonal communication about contraception has been developed and validated^46, 47^ and recently endorsed by the National Quality Forum as a system-level performance measure.^48^ This measure provides an opportunity for VA to collect data on interpersonal quality of communication on an ongoing basis to aid efforts to improve contraceptive counseling quality. An additional limitation was the small numbers of non-heterosexual women included in the sample (*n*=31), limiting our ability to adequately explore sexual orientation as a factor that could influence experience. Furthermore, ECUUN did not capture gender identity despite the fact that some Veterans who can become pregnant do not identify as women. Studies outside of VA have found LGBTQ individuals face unmet contraceptive need^45, 46^ and barriers to high-quality contraceptive care,^47^ indicating a need for additional attention to the needs of these populations.

In conclusion, the provision of high-quality, patient-centered contraceptive care is an essential component of primary care and critical to ensure that all Veterans receiving care at VA can make informed decisions about their contraceptive options and achieve their reproductive desires and goals. While the VA has made great strides in improving the quality of reproductive health services for Veterans, continued efforts are needed to ensure that high-quality contraceptive counseling is consistently delivered across primary care settings.
